# Effects of polygenic risk for Alzheimer’s disease on rate of cognitive decline in normal aging

**DOI:** 10.1038/s41398-020-00934-y

**Published:** 2020-07-24

**Authors:** Karolina Kauppi, Michael Rönnlund, Annelie Nordin Adolfsson, Sara Pudas, Rolf Adolfsson

**Affiliations:** 1 Department of Integrative Medical Biologi, Umeå University, Umeå, Sweden; 2 Department of Medical Epidemiology and Biostatistics, Karolinska Institutet, Stockholm, Sweden; 3 Department of Psychology, Umeå University, Umeå, Sweden; 4 Department of Clinical Sciences, Umeå University, Umeå, Sweden

**Keywords:** Medical genetics, Learning and memory

## Abstract

Most people’s cognitive abilities decline with age, with significant and partly genetically driven, individual differences in rate of change. Although APOE ɛ4 and genetic scores for late-onset Alzheimer’s disease (LOAD) have been related to cognitive decline during preclinical stages of dementia, there is limited knowledge concerning genetic factors implied in normal cognitive aging. In the present study, we examined three potential genetic predictors of age-related cognitive decline as follows: (1) the APOE ɛ4 allele, (2) a polygenic score for general cognitive ability (PGS-cog), and (3) a polygenic risk score for late-onset AD (PRS-LOAD). We examined up to six time points of cognitive measurements in the longitudinal population-based Betula study, covering a 25-year follow-up period. Only participants that remained alive and non-demented until the most recent dementia screening (1–3 years after the last test occasion) were included (*n* = 1087). Individual differences in rate of cognitive change (composite score) were predicted by the PRS-LOAD and APOE ɛ4, but not by PGS-cog. To control for the possibility that the results reflected a preclinical state of Alzheimer’s disease in some participants, we re-ran the analyses excluding cognitive data from the last test occasion to model cognitive change up-until a minimum of 6 years before potential onset of clinical Alzheimers. Strikingly, the association of PRS-LOAD, but not APOE ɛ4, with cognitive change remained. The results indicate that PRS-LOAD predicts individual difference in rate of cognitive decline in normal aging, but it remains to be determined to what extent this reflects preclinical Alzheimer’s disease brain pathophysiology and subsequent risk to develop the disease.

## Introduction

In normal aging, a pattern of decline is observed across a multitude of cognitive domains, although the magnitude differs across domains and individual differences in rate of change are substantial^1–3^. Although some older individuals resist cognitive decline over test occasions separated by decades^3^, others progress to clinical dementia. Late-onset Alzheimer’s disease (LOAD), the most common form of dementia, is a slowly progressive disorder typically manifested by a preclinical phase with gradual decline in cognitive and social abilities years before the criteria for a clinical diagnosis are met^4–6^. Due to a high societal disease burden of Alzheimer’s disease, much research is focused on prediction models that can separate early stages of Alzheimer’s disease from normal cognitive aging^7–11^. However, also within the range of cognitive impairment considered as normal, cognitive aging has a large impact on individuals’ functional outcome and independence^12^. The exact mechanisms underlying normal cognitive aging are poorly understood, but are oftentimes considered distinct from Alzheimer’s disease and other neurocognitive diseases affecting the elderly^12^.

Between-person variability in level of cognitive functioning is largely dependent on genetic factors, with twin heritability estimates ranging from 50 to 80%^13,14^ and modest differences in heritability of cognitive level at different age groups^15^. Few large-scale twin studies have been able to estimate the heritability of cognitive change, but one study based on 798 non-demented twins followed over 13 years reported substantial heritability of rate of change in cognitive performance, although generally lower than for cognitive level^13^. There was no link between the heritability of intercept and slope, indicating that the genetic factors influencing level and change in cognitive ability may not be the same^15^. Heritability estimates from molecular genetic studies based on common single-nucleotide polymorphisms (SNPs) are typically lower than twin-based estimates and have varied from 5% for level of memory function to 31% for level of verbal-numeric reasoning^15,16^. The few SNP-based heritability estimates of change in general cognitive ability available amount to about 25%^15^. As few studies involved large-scale longitudinal data on cognitive aging and genetics, the specific genetic factors underlying normal cognitive aging remain to be further investigated. Currently, it is unknown whether there is genetic overlap between normal age-related cognitive decline and Alzheimer’s disease.

LOAD is considered as the most heritable dementia subtype, with twin heritability estimates of 58–79%^17^. Although APOE ɛ4 accounts for a large proportion of the genetic effect on Alzheimer’s disease risk, at least half of the heritability has been shown to be represented by a large number of genes not located at chromosome 19, harboring APOE; moreover, the importance of APOE decreases with increased age at disease onset^18^. Using the PRS/PGS approach, one can examine genetic association between traits and endophenotypes, which is helpful to reveal potentially overlapping biological mechanisms^19,20^. For each individual, the cumulative effect of a large number of genetic variants across the whole genome is then estimated by summing each variant’s effect size on the target trait from a previous genome-wide association study (GWAS) multiplied by the number of effect alleles (0,1,2) in that individual^19,20^.

In the present study, we examined genetic predictors of cognitive level and change based on a longitudinal data set spanning a 25-year period. We hypothesized that change in cognitive ability in normal aging is influenced either by genes related to level of cognitive ability and/or genes related to Alzheimer’s disease. We considered two polygenic predictors of interest as follows: (1) a PRS for Alzheimer’s Disease (PRS-LOAD, based on IGAP data^21^, *n* = 74,046) and (2) a PGS for cognitive performance, (PGS-Cog, based on data from a meta-analysis of COGENT^22^ and the UK Biobank, performed by Lee et al^23^, *n* = 257,841). In addition, we examined the effect of the APOE ε4 allele alone. As the focus was to examine the genetic underpinning of normal cognitive aging, we only included individuals that were non-demented at the last test occasion and remained non-demented up until the most recent dementia screening, conducted between one to three years after the last test occasion. The cognitive trajectories of the included participants in our well-screened sample thus reflect age-related cognitive decline in healthy elderly.

## Methods

### Sample and participants

Data emanated from the longitudinal Betula study, a prospective population-based cohort study of aging and dementia conducted in Sweden^24,25^. Extensive health- and cognitive data were collected over six test waves (T1–T6) five years apart, with a total follow-up period of 25 years^24,25^. A total of 1481 participants from the cohorts S1 and S3, aged 35–80 years, who where non-demented at inclusion and had been successfully genotyped were included. To focus on cognitive level and change in normal cognitive aging, all participants that subsequently developed dementia up until the last dementia screening (2015–2017) were excluded from the main analyses (*n* = 283). These included the following dementia subtypes: dementia of the Alzheimer’s type (*n* = 152), vascular dementia (*n* = 102), dementia NOS (*n* = 13), dementia due to Parkinson’s disease (*n* = 8), Lewy body dementia (*n* = 5), frontotemporal dementia (*n* = 2), and progressive supranuclear paralysis (*n* = 1). Participants with unknown dementia status due to moving from the area, not giving their permission for researchers to view their medical records, or insufficient information to draw firm diagnostic conclusions were also excluded (*n* = 111). For the remaining sample of 1087 subsequently non-demented individuals (577 females and 510 males), longitudinal cognitive data were available for the following follow-up time: 5 years; *n* = 985, 10 years; *n* = 827, 15 years: *n* = 611, 20 years: *n* = 397, 25 years: *n* = 143 (see Supplementary Table S1 for age distribution by time from inclusion). All participants gave their written informed consent for participation.

### Dementia diagnosis procedure

Dementia diagnoses were based on the DSM-IV criteria^26^ and were determined through a process based on multiple sources of information where thorough evaluation of regional medical records from virtually all clinical disciplines formed the basis, allowing a chronology of medical history, other clinicians’ assessments, and results from available neuroradiological examinations to be integrated in the assessment. Contributory information was obtained from the Betula study health examinations and neuropsychological test assessments. Particular attention was given to participants who met one or several of the following pre-defined criteria; low score (≥1.8 SDs below age-based norms) on a composite cognition and memory test, with a decline in cognitive performance from a previous test occasion (from high to average or low, or from average to low); a low score (≤23) or a drop by 3 points, compared with previous score, on the Mini-Mental State Examination^27^; self-reported memory impairment or evident clinical signs of neurocognitive dysfunction observed in the test situations. To increase the diagnostic precision and the reliability of the assessments, diagnostic evaluations were performed repeatedly, with start at baseline and thereafter every 5 years. At each diagnostic follow-up, medical records were studied without the responsible psychogeriatric study physician having access to previous status. Potential conflicts between the recent determined and the previously established diagnosis resulted in a further investigation to establish a conclusive status. The dementia subtypes Alzheimer’s disease and vascular dementia were, beyond symptoms attributable to either of those dementias, characterized by a progressive decline. Disease onset was defined as the time at which the clinical symptoms became sufficiently severe to interfere with social functioning and instrumental activities of daily living, i.e., when the core criteria for dementia were met^28^. The diagnostic evaluation was coordinated by the research geropsychiatrist (co-author R.A.) throughout the study period.

### Cognitive tests

To maximize power, we based the main analyses on a *z*-transformed cognitive composite score, Cog-Comp, which was calculated as the sum of *z*-transformed test of episodic recall, vocabulary, block design, and verbal fluency (*n* = 1081 with cognitive data). Thereafter, additional complementary analyses were performed separately, for each of the cognitive domains. Below follows a short description of each of those four measures, which are described in more detail in refs. ^24,25^.

For episodic memory, a composite score (Mem-comp) based on the sum of recalled items of five different tests served as the dependent measure^29^. The first two test consisted of free oral recall of 16 verb-noun sentences encoded with or without enactment. The stimuli were presented at a rate of 8 s/item with a 2 min time limit for recall. Thereafter, two subsequent tests consisted of category-cued recall of nouns from the sentence recall and the action recall. The fifth test was a free recall test of 12 nouns that were presented at a pace of 2 s/word with a 45 s time limit for free recall. The Block Design task was adopted from the Wechsler Adult Intelligence Scale (WAIS-R), as a measure of visuospatial constructional ability. The task consists of arranging a set of four black and white blocks as fast as possible according to a given pattern, with ten target patterns included^30^. The vocabulary test required that the participant identified the synonym for a list of words (*n* = 30) among five alternatives. For verbal fluency, we used the sum of three measures that required the verbal generation of as many words (names not allowed) as possible during 1 min given the following restrictions: (1) words beginning with the letter A, (2) words with the initial letter M and containing five letters, and (3) professions with initial letter B. The number of correctly generated words was used as the dependent measure.

### Polygenic scores

The majority of DNA was extracted for SNPs genotyping at VIB-U Antwerp Center for Molecular Neurology in Belgium (89%). A fraction of samples was extracted at Genome-wide Genotyping LGC Genomics Ltd, UK (18%). All individuals were genotyped using two types of Illumina Infinium arrays: The Infinium Human OmniExpress-12v1_H and the Infinium Exome Array. The genotyping was conducted at the Genotyping Platform of the Broad Institute of MIT and Harvard, USA, between 2012 and 2014. Raw genotypes were imputed towards the 1000 Genome using the imputation pipeline ricopili used by the psychiatric genomic consortia^31^. Imputed best-guess genotypes with a genotyping call probability of >0.8 were used for generation of scores. First, post-imputation quality control was performed based on minor allele frequency < 1 %. No individuals had a genotyping call rate lower than 10%, and no SNPs had a missingness under 5%. PRS/PGS were then calculated after removal of SNPs with ambiguous strand alignment, using the profile score function in PLINK 1.9. First, linkage-disequilibrium clumping was performed using a *r*^2^ > 0.1 threshold over 250 kb sliding windows (using a threshold for index and clumped SNPs of *p* < 1). Each polygenic score was calculated as the sum of effective allele count (0,1,2) × *β*-value from publicly available summary statistics from published GWAS studies on cognitive performance^23^ or Alzheimer’s disease^21^, for PGS-Cog and PRS-LOAD respectively. For calculation of PRS-LOAD, we used summary statistics from a meta-analysis of four previous GWAS of LOAD containing 17,008 Alzheimer’s disease patients and 37,154 controls (stage 1)^21^. For the main analyses, we used scores including all SNPs from the GWAS summary statistics (*p* < 1). As a sensitivity analysis, we also calculated nine complementary scores using the following *p*-value thresholds: 0.5, 0.4, 0.3, 0.2, 0.1, 0.05, 0.01, 0.001, and 0.0001. In addition, we also calculated a PRS-LOAD where the APOE loci was removed (removing all SNPs within chr 19: 44,400–46,500 Mb).

### Statistical analyses

To examine the association of PGS/PRSs with level and change in cognitive measures over 25 years, we performed linear mixed-effect models. Mixed-effect models were fitted via maximum likelihood in R (version 3.5.1) using the lmer function available through the lme4 package. *P*-values were estimated based on the Satterthwaite approximations implemented in the lmerTest packages^32^. For model build, we first ran a full model with the following variables: Sample, age, age^2^, sex, APOE ɛ4, and APOE ɛ2, the ten first principal components for genetic ancestry (to control for population stratification)^33^, and the two polygenic scores of interest (Supplementary Table S2). Nonsignificant variables were then hierarchically removed one by one starting with the one with highest *p*-value, until only the variables with at least trend significance on either intercept or slope remained in the model (*p* < 0.06). All our three genetic predictors of interest were thereafter included to the same model, including the following significant variables of no interest: Age, Age^2^, Sex, and genetic principal components for genetic ancestry 1–3 and 6. Years from inclusion was used as time-scale, i.e., to represent slope, and interaction with time was allowed for all covariates (see full model in Supplementary Table S2). The model allowed for random subject-specific intercept.

As APOE ɛ4 constitutes a substantial proportion of Alzheimer’s disease genetics, we also examined whether PRS-LOAD was predictive over and above APOE ɛ4, by model comparisons, where a model with only APOE ɛ4 was compared with a model including both APOE ɛ4 and PRS-LOAD, using analysis of variance in R. As a sensitivity analysis, we re-ran the main analyses using the PRS-LOAD calculated without the APOE loci.

For descriptive purposes, the relationship between age and the Cog-Comp was estimated with a generalized additive mixed model (GAMM)^34^ with the gamm4 R package (GAMMs using mgcv and lme4. R package version 0.2-3. http://CRAN.R-project.org/package=gamm4, by Wood and Scheipl).

## Results

### Cognitive performance descriptive and covariates

**Fig. 1:**
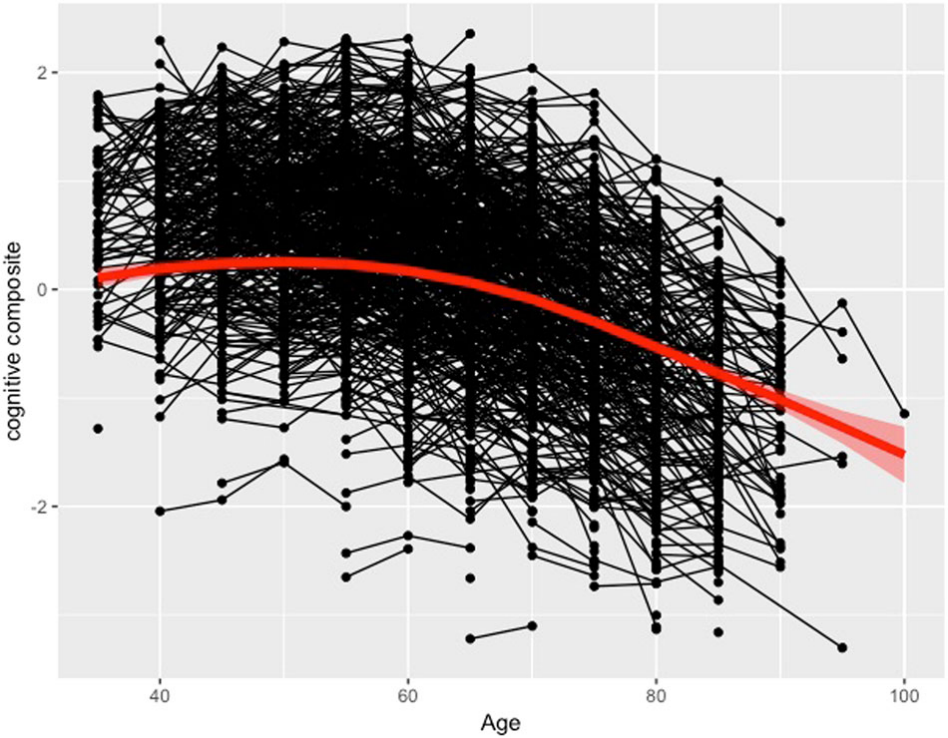
Individual trajectories in cognitive performance. Spaghetti plot of individual trajectories in cognitive performance (*Z*-transformed cognitive composite score) across 20 years, as well as the population-average trend (red line), estimated with a generalized additive mixed model.

A plot of the GAMM model describing the average cognitive trajectory over time shows that cognitive function on average remains stable until age 60 years, where after performance drops (Fig. 1), which also motivates inclusion of a quadratic age covariate in the linear models. Descriptive statistics for each cognitive test and test occasion (T1–T6) of the study are shown in Supplementary Table S3. The following variables of no interest were significantly associated to intercept and/or slope in the linear mixed-effect model and thus included as covariates in the main analyses of the genetic predictors; Age, age^2^, genetic principal components 1–3 and 6, and sex (Supplementary Table S2, Sex at trend level).

### Genetic prediction of level and change in cognitive performance

Using linear mixed-effect models, we examined the association of PGS-Cog, PRS-LOAD, and APOE ɛ4 on the level and slope of cognitive performance. All genetic predictors were entered to the same model together with covariates of no interest as stated above. Level of cognitive performance was strongly predicted by the PGS-Cog (*p* = 6.04e − 13, uncorrected, Table 1), but not by PRS-LOAD or APOE ɛ4. A significant negative association with slope was seen for both PRS-LOAD (*p* = 0.018 uncorrected) and APOE ɛ4 (*p* = 0.043, uncorrected), whereas no effect was seen for the PGS-Cog (Table 1). Model comparisons showed that inclusion of the AD-PRS to a model with only APOE ɛ4 significantly improved the model (*χ*^2^ = 7.33 (2), *p* = 0.026). Including the three genetic predictors individually in separate models revealed similar effects on level and slope, with somewhat larger effect of APOE ɛ4 and PRS-LOAD on cognitive slope (APOE ɛ4: *β* = −3.46e − 3(1.68e − 3), *p* = 0.0389, PRS-LOAD: *β* = −1.82e − 3(7.65e − 4), *p* = 0.0169).

**Table 1:** Linear mixed model of association with the cognitive composite score (cog-comp).

	Estimate	SE	*t*	*p*	
(Intercept)	2.622e − 01	7.640e − 02	3.432	0.000621	***
Age at inclusion	−5.646e − 01	2.354e − 02	−23.988	2.00E − 16	***
Time	−1.952e − 02	2.497e − 03	−7.815	7.82e − 15	***
Age^2^	−1.496e − 01	2.427e − 02	−6.167	9.58e − 10	***
Sex	−7.792e − 02	4.534e − 02	−1.718	0.085975	.
C1	9.926e − 02	2.283e − 02	4.347	1.50e − 05	***
C2	4.810e − 02	2.308e − 02	2.084	0.037368	*
C3	8.159e − 02	2.298e − 02	3.551	0.000399	***
C6	−4.455e − 02	2.292e − 02	−1.944	0.052108	.
APOE ɛ4	−9.845e − 03	5.150e − 02	−0.191	0.848433	
**PGS-COG**	1.677e − 01	2.303e − 02	7.283	**6.04e−13**	***
PRS-LOAD	−1.968e − 02	2.312e − 02	−0.851	0.394875	
Time × age	−1.626e − 02	1.205e − 03	−13.495	2.00E − 16	***
Time × age inclusion^2^	2.185e − 03	1.063e − 03	2.056	0.039828	*
Time × sex	−2.898e − 03	1.503e − 03	−1.928	0.053918	.
Time × C1	7.254e − 04	7.440e − 04	0.975	0.329656	
Time × C2	8.406e − 04	7.402e − 04	1.136	0.256244	
Time × C3	−1.020e − 03	7.617e − 04	−1.339	0.180840	
Time*C6	1.003e − 03	7.673e − 04	1.307	0.191263	
**Time×APOE ɛ4**	−3.394e − 03	1.677e − 03	−2.024	**0.043062**	*
Time × PGS-COG	−3.240e − 04	7.858e − 04	−0.412	0.680098	
**Time×PRS-LOAD**	−1.811e − 03	7.642e − 04	−2.370	**0.017839**	*

*n* = 1081 individuals that remained non-demented up until 1 year after the last cognitive test occasion. All continuous variables are scaled. Slope estimated across up to six time points; T1–T6. *p = <0.05, **p = <0.01, ***p = 0.001. All *p*’s are reported at an uncorrected level.

Bold values indicates significant variables of interest.

Next, to reduce the potential influence on slope by individuals in a potential preclinical stage of Alzheimer’s disease, we re-ran the model without the last test occasion. This analysis modeled the genetic effect on cognitive slope up until a minimum of 6 years before the last dementia assessment, when the included individuals were confirmed to have stayed non-demented. In these analyses, the effect of the PRS-LOAD remained significant (*p* = 0.0232), whereas APOE ɛ4 did not (Table 2). Again, model comparisons showed that adding PRS-LOAD on top of APOE ɛ4 significantly improved the model (*χ*^2^ = 6.85 (2), *p* = 0.0326). A model with APOE ɛ4 as the only genetic variable included was not significant (*p* = 0.0938) and adding APOE ɛ4 on top of a model without genetic predictors did not improve the model significantly (*χ*^2^ = 2.85(2), *p* = 0.2). The effect of PRS-LOAD was again somewhat larger without other genetic variables included in the same model (*β* = −2.04e − 3(8.74e − 4), *p* = 0.0197).

**Table 2:** Effect of APOE ɛ4 and the two polygenic scores on cognitive performance (cog-comp).

T1–T5		Estimate	SE	*t*	*p*	
	APOE ɛ4	−1.198e − 02	5.167e − 02	−0.232	0.816708	
	**PGS-COG**	1.697e − 01	2.310e − 02	7.346	**3.82e−13**	***
	PRS-LOAD	−1.915e − 02	2.320e − 02	−0.825	0.409343	
	Time*APOE ɛ4	−2.985e − 03	1.932e − 03	−1.545	0.122389	
	Time*PGS-COG	−9.061e − 04	9.045e − 04	−1.002	0.316550	
	Time*PRS-LOAD	−1.985e − 03	8.739e − 04	−2.271	**0.023207**	*

Effect of APOE ɛ4 and the two polygenic scores on cognitive performance (cog-comp), estimated based on cognitive tests performed up until T5, when participants were subsequently confirmed to have stayed non-demented for at least 6 years. *n* = 1081 participants. *p = <0.05, **p = <0.01, ***p = 0.001. All *p*’s are reported at an uncorrected level.

Bold values indicates significant variables.

For completeness, we re-ran the main analyses using a PRS-LOAD where the APOE ɛ4 loci had been removed. Those results remained highly similar, with a significant effect of PRS-LOAD without APOE on cognitive slope both estimated using all test occasions (*β* = −1.83 − 3(7.64e − 4), *p* = 0.018) and without the last test occasion (*β* = −1.98(8.74e − 4), *p* = 0.024).

Additional analyses presented in the supplementary include the following: (1) genetic prediction of the level and slope of individual cognitive test performance (Supplementary Table S4 and Supplementary Fig. S1), most importantly showing the strongest effect of PRS-LOAD on slope of verbal fluency and block design; (2) genetic prediction of level and slope of cognitive performance irrespective of dementia status, mainly showing enlarged effect of APOE and PRS-LOAD on slope, and (3) control analyses using different *p*-value thresholds for polygenic scores, with similar results (Supplementary Table S5).

## Discussion

In this 25-year longitudinal data set of non-demented individuals aged 35–80 years at inclusion, genetic predictors of normal age-related cognitive decline were evident. We found that age-related cognitive decline was predicted by higher genetic risk for Alzheimer’s disease but not by a genetic profile score for overall cognitive performance level. Importantly, polygenic risk for Alzheimer’s disease remained a significant predictor of cognitive decline also when modeling slope without the last test occasion, which reduces the influence by participants in a preclinical phase of Alzheimer’s disease 6 years or less before clinical disease onset. Notably, the effect of PRS-LOAD was stronger than that for APOE ɛ4 alone and APOE ɛ4 had no significant effect on the slope modeled without the last test occasion. The present results suggest a genetic overlap between normal age-related cognitive decline and genetics of Alzheimer’s disease.

Previous studies have examined genetic predictors of cognitive decline primarily in individuals in a preclinical stage of Alzheimer’s disease, defined from the occurrence of brain atrophy or abnormal CSF biomarkers of Alzheimer’s disease, and found significant effects of APOE ɛ4^35^ and polygenic scores weighted on association to Alzheimer’s disease^35,36^, on episodic memory^37^. However, two previous studies based on the Lothian birth cohort also focused on genetic predictors of normal cognitive aging, but failed to identify an effect of polygenic risk for Alzheimer’s disease on age-related cognitive decline^38,39^, although a significant effect of APOE ɛ4 was observed on cognitive slope^39^. Compared with those studies, we used data from a sample with a longer follow-up time, more measurement points, and a wider age range, where the previous study only estimated cognitive change between age 70 and 79 years.

Alzheimer’s disease is a slowly progressive disorder characterized by a long preclinical phase with gradually increasing levels of Alzheimer’s disease -related pathological processes, including amyloid, tau and brain atrophy^40^, and cognitive decline, starting up to a decade prior to disease onset^40,41^. Thus, if the variation in cognitive decline observed in our study group to some extent reflect early preclinical stage of Alzheimer’s disease (>6 years before clinical onset), the observed effect of the genetic score may be a result of a link to Alzheimer’s disease rather than normal cognitive aging. In that case, the results suggest that the PRS-LOAD predicts cognitive decline over and above APOE ɛ4 at a very early stage of AD progression. Thus, the genetic score may thus be useful for early prediction of at-risk individuals, and also to understand the mechanisms of early clinical manifestations of Alzheimer’s disease. In line with an effect of PRS-LOAD on early disease processes, a recent study showed that a PRS for Alzheimer’s disease was associated with increased odds ratio of having mild cognitive impairment compared to normal controls already at the age of 50 years, i.e., decades before typical onset of Alzheimer’s disease^42^.

An alternative possibility is that the current findings indicate that the biological processes underlying cognitive decline in normal aging to some extent overlap with the pathological processes of Alzheimer’s disease. Recently, there has been a diagnostic shift, where an Alzheimer’s disease diagnosis can now be confirmed based on the combination of clinical symptoms and presence of Alzheimer’s specific pathology, most importantly amyloid and neurodegeneration^40,43^. Age-related cognitive decline prior to clinical dementia onset has been linked to various measures of Alzheimer’s disease pathology^5,44–46^. However, some studies found no such link^47^ and Alzheimer’s disease pathology have also been seen in individuals without cognitive symptoms and is not deterministic of future disease progression^40,46^. As most of these studies focused on preclinical dementia or healthy samples with lack of diagnostic follow-up, there are no definite answers to what extent Alzheimer’s disease-specific pathology may contribute to cognitive decline also in elderly who will not subsequently develop Alzheimer’s disease. PRS-AD has also been associated with level of cognitive test performance from cross-sectional studies, e.g., in the UK Biobank where tests of verbal-numeric reasoning and memory were assessed on 36,035 and 112,067 participants, respectively^48^. As individuals up to 73 years old were included, without follow-up on development of dementia, it is difficult to assess to what extent those results may represent preclinical stages of AD development or a genetic overlap between AD and cognition in healthy.

In addition to the main findings, we extended previous findings of a strong association of PGS-Cog to the level of cognitive performance in young and middle age^39^, to an older population. This result suggests that genetic factors of cognitive ability remain stable across the adult lifespan, while the genetic underpinnings of cognitive level and slope are different. Another notable finding was that APOE ɛ4 predicted slope but not level of cognitive performance in this study group, and that the effect on slope was only seen when including the last test occasion where the probability of individuals being at a preclinical stage of Alzheimer’s disease was higher. This result strongly implies the PRS-LOAD as a more sensitive predictor of cognitive decline than APOE ɛ4 in normal aging. The same pattern was seen in the above-mentioned study from the Lothian birth cohort, where APOE ɛ4 also had a strong effect on cognitive slope, and only marginal effect on cognitive intercept^39^. This may be due to higher predictive power of the polygenic score or indicate that APOE ɛ4 is specifically related to risk for Alzheimer’s disease through pathological processes that are not involved in normal aging, or impact later stages of the disease progression.

### Limitations

Although the participants were randomly drawn from the population registry initially, attrition rate has been shown to be non-random^49^. Lower rates of dropouts are seen among healthier individuals, those that were younger at inclusion, and those with higher cognitive level at baseline. Individuals with the largest number of measurement points for calculation of cognitive slope are among those with the lowest risk to develop Alzheimer’s disease, which may bias the results toward a healthier sample than the general population^49^. As we aimed to study healthy cognitive aging, this bias would if anything strengthen the validity of the results. It should also be noted that the Alzheimer’s disease diagnoses in this study were not based on biomarkers or neuropathological examinations, but instead reflect “clinical Alzheimer’s disease,” based on careful review of medical records and information from cognitive and health tests within the study. To minimize the risk of misclassification, diagnoses were validated by repeated assessments every five years.

## Conclusions

We found that a PRS-LOAD predicted rate of cognitive decline in a well-screened sample of healthy older adults who remained non-demented up until at least six years after last assessment, over and beyond the APOE ɛ4 allele. Such genetic overlap between healthy cognitive aging and Alzheimer’s disease suggest that cognitive decline observed in a general non-demented population is at least in part linked to Alzheimer’s disease-related pathologies more than six years before potential clinical onset of dementia. The current data cannot reveal whether the observed genetic overlap represents cognitive decline in individuals at an early preclinical disease stage, or whether cognitive decline in individuals that will not eventually develop Alzheimer’s disease is also partly influenced by biological processes related to this disease. Continuous long-term follow-up of those individuals in respect to potential development of dementia is warranted. In addition, collection of biomarkers of Alzheimer’s disease could reveal to what extent the genetic link to cognitive decline in this study cohort is mediated by Alzheimer’s disease-related pathologies.

## Supplementary information

Supplementary materials

## References

[1] Craik, F. I. M. & Salthouse, T. A. *The Handbook of Aging and Cognition: Third Edition*. 1st ed. (Psychology Press, New york, 2008).

[2] Wilson RS (2002). Individual differences in rates of change in cognitive abilities of older persons. Psychol. Aging.

[3] Nyberg L, Pudas S (2019). Successful Memory Aging. Annu Rev. Psychol..

[4] Price JL (2009). Neuropathology of nondemented aging: Presumptive evidence for preclinical Alzheimer disease. Neurobiol. Aging.

[5] Hanseeuw BJ (2019). Association of amyloid and tau with cognition in preclinical Alzheimer disease: a longitudinal study. JAMA Neurol..

[6] Belleville S, Fouquet C, Duchesne S, Collins DL, Hudon C (2014). Detecting early preclinical alzheimer’s disease via cognition, neuropsychiatry, and neuroimaging: qualitative review and recommendations for testing. J. Alzheimer’s Dis..

[7] Kauppi K (2018). Combining polygenic hazard score with volumetric MRI and cognitive measures improves prediction of progression from mild cognitive impairment to Alzheimer’s disease. Front Neurosci..

[8] Dukart J, Sambataro F, Bertolino A (2015). Accurate prediction of conversion to Alzheimer’s disease using imaging, genetic, and neuropsychological biomarkers. J. Alzheimer’s Dis..

[9] Teipel, S. J. et al. Predictors of cognitive decline and treatment response in a clinical trial on suspected prodromal Alzheimer’s disease. *Neuropharmacology* **108**, 128–135 (2016). 10.1016/j.neuropharm.2016.02.005 26876309

[10] Vemuri P (2009). MRI and CSF biomarkers in normal, MCL and AD subjects: predicting future clinical change. Neurology.

[11] Chatterjee N, Shi J, García-Closas M (2016). Developing and evaluating polygenic risk prediction models for stratified disease prevention. Nat. Rev. Genet..

[12] Blazer DG, Yaffe K, Karlawish J (2015). Cognitive aging: a report from the Institute of Medicine. JAMA.

[13] Finkel D, Pedersen NL (2004). Processing speed and longitudinal trajectories of change for cognitive abilities: The Swedish Adoption/Twin Study of Aging. Aging Neuropsychol. Cogn..

[14] McClearn GE (1997). Substantial genetic influence on cognitive abilities in twins 80 or more years old. Science (80).

[15] Reynolds CA, Finkel D (2015). A meta-analysis of heritability of cognitive aging: minding the “missing heritability” gap. Neuropsychol. Rev..

[16] Davies G (2016). Genome-wide association study of cognitive functions and educational attainment in UK Biobank (N = 112151). Mol. psy.

[17] Gatz MS (2006). Role of genes and environments for explaining Alzheimer disease. Arch. Gen. Psychiatry.

[18] Lo, M. et al. Identification of genetic heterogeneity of Alzheimer’s disease across age. *Neurobiol Aging* 2019; in press. 10.1016/j.neurobiolaging.2019.02.022 PMC6783343 30979435

[19] Chasioti D, Yan J, Nho K, Saykin AJ (2019). Progress in polygenic composite scores in Alzheimer’s and other complex diseases. Trends Genet..

[20] Martin AR, Daly MJ, Robinson EB, Hyman SE, Neale BM (2019). Predicting polygenic risk of psychiatric disorders. Biol. Psychiatry.

[21] Lambert J-C (2013). Meta-analysis of 74,046 individuals identifies 11 new susceptibility loci for Alzheimer’s disease. Nat. Genet.

[22] Trampush JW (2017). GWAS meta-analysis reveals novel loci and genetic correlates for general cognitive function: a report from the COGENT consortium. Mol. Psychiatry.

[23] Lee JJ (2018). Gene discovery and polygenic prediction from a genome-wide association study of educational attainment in 1.1 million individuals. Nat. Genet.

[24] Nilsson L-G (1997). The betula prospective cohort study: memory, health, and aging. Aging, Neuropsychol. Cogn..

[25] Nilsson L-G (2004). Betula: a prospective cohort study on memory, health and aging. Aging Neuropsychol. Cogn..

[26] American Psychiatric Association. *Diagnostic and Statistical Manual of Mental Disorders*. 4th edn (American Psychiatric Press Ink, Washington DC, 2000).

[27] Folstein MF, Folstein SE, McHugh PR (1975). Mini-mental state”: a practical method for grading the cognitive state of patients for the clinician. J. Psychiatr. Res..

[28] McKhann GM (2011). The diagnosis of dementia due to Alzheimer’s disease: recommendations from the National Institute on Aging-Alzheimer’s Association workgroups on diagnostic guidelines for Alzheimer’s disease. Alzheimer’s Dement.

[29] Rönnlund M, Nilsson L-G (2006). The Betula Study: reliabilities and long-term stabilities of memory test performances over the adult lifespan. Balt. J. Psychol..

[30] Rönnlund M, Nilsson L-G (2006). Adult life-span patterns in WAIS-R Block Design performance: cross-sectional versus longitudinal age gradients and relations to demographic factors. Intelligence.

[31] Lam M (2019). RICOPILI: Rapid Imputation for COnsortias PIpeLIne. Bioinformatics.

[32] Luke SG (2017). Evaluating significance in linear mixed-effects models in R. Behav. Res Methods.

[33] Price AL (2006). Principal components analysis corrects for stratification in genome-wide association studies. Nat. Genet.

[34] Woods S. N. *Generalized Additive Models: An introduction with R*. 2nd edn (CRC Press, Boca Raton, 2006).

[35] Porter T (2018). Utility of an Alzheimer’s disease risk-weighted polygenic risk score for predicting rates of cognitive decline in oreclinical Alzheimer’s disease: a Prospective Longitudinal Study. J. Alzheimer’s Dis..

[36] Ge T, Sabuncu MR, Smoller JW, Sperling RA, Mormino EC (2018). Dissociable influences of APOE «4 and polygenic risk of AD dementia on amyloid and cognition. Neurology.

[37] Porter T (2018). A polygenic risk score derived from episodic memory weighted genetic variants is associated with cognitive decline. Preclinical Alzheimer’ s. Dis..

[38] Harris SE (2014). Polygenic risk for alzheimer’s disease is not associated with cognitive ability or cognitive aging in non-demented older people. J. Alzheimer’s Dis..

[39] Ritchie, S. J. et al. Polygenic predictors of age-related decline in cognitive ability. *Mol. Psychiatry*10.1038/s41380-019-0372-x (2019). 10.1038/s41380-019-0372-x PMC7515838 30760887

[40] Scheltens P (2016). Alzheimer’ s disease. Lancet.

[41] Niemantsverdriet E, Valckx S, Bjerke M, Engelborghs S (2017). Alzheimer’s disease CSF biomarkers: clinical indications and rational use. Acta Neurol. Belg..

[42] Logue MW (2019). Use of an Alzheimer’s disease polygenic risk score to identify mild cognitive impairment in adults in their 50s. Mol. Psychiatry.

[43] Jack CR (2019). Prevalence of biologically vs clinically defined Alzheimer spectrum entities using the National Institute on Aging-Alzheimer’s Association Research Framework. JAMA Neurol..

[44] Resnick SM (2010). Longitudinal cognitive decline is associated with fibrillar amyloid-beta measured by [^11^C]PiB. Neurology.

[45] Bilgel M (2018). Effects of amyloid pathology and neurodegeneration on cognitive change in cognitively normal adults. Brain.

[46] Jack CR (2019). Associations of amyloid, tau, and neurodegeneration biomarker profiles with rates of memory decline among individuals without dementia. JAMA.

[47] Kapasi A, DeCarli C, Schneider JA (2017). Impact of multiple pathologies on the threshold for clinically overt dementia. Acta Neuropathol..

[48] Hagenaars SP (2016). Shared genetic aetiology between cognitive functions and physical and mental health in UK Biobank (N=112 151) and 24 GWAS consortia. Mol. Psychiatry.

[49] Josefsson M, De Luna X, Pudas S, Nilsson LG, Nyberg L (2012). Genetic and lifestyle predictors of 15-year longitudinal change in episodic memory. J. Am. Geriatr. Soc..

