# Supplementary material for: Effects of polygenic risk for Alzheimer’s disease on rate of cognitive decline in normal aging
Source: Transl Psychiatry. 2020 Jul 24;10:250. doi: 10.1038/s41398-020-00934-y (PMC7381667; doi:10.1038/s41398-020-00934-y)

Supplementary materials for

Effects of polygenic risk for Alzheimer´s disease on rate of cognitive decline in normal aging

Karolina Kauppi, Michael Rönnlund, Annelie Nordin Adolfsson, Sara Pudas, Rolf Adolfsson

***S*upplementary results**

***Genetic prediction of level and slope of individual cognitive test performance***

Analyses of individual tests showed that APOE ɛ4 did not significantly predict level or slope of any test. PGS-Cog was predictive of level of all tests, with the strongest effect on vocabulary (all p’s<5e-5), with no effect on the slope of any test. PRS-LOAD did not predict level of any individual test. PRS-LOAD significantly predicted the slope of verbal fluency (p=0.036) and block design (p=0.037), and a non-significant trend in the same direction of was seen for episodic memory. All results are presented in **Table S3**).

***Genetic prediction of level and slope of cognitive performance irrespective of dementia status***

For completeness, we also run the above analyses including also individuals with a subsequent Alzheimer’s diagnosis (N=152), using all six test occasions. Again, a significant effect of PGS-Cog was seen on level (β=1.638e-1 (2.192e-02), p= 1.45e-13) but not slope of the cognitive composite score. Also in this group, PRS-LOAD and APOE ɛ4 had no effect on cognitive level, whereas a larger effect was seen on cognitive slope (APOE ɛ4: β=-7.34e-3(1,68e-3), p= 1,27e-5, PRS-LOAD: β=-2,62 e-3(7,84e-4), p=0.00082). Model comparisons revealed a significantly improved prediction when adding PRS-LOAD on top of only APOE ɛ4 (χ^2^=11.68, p=0.0029).

***Control analyses using different p-value thresholds for polygenic scores***

For the main analyses, we used a p-value threshold for inclusion of SNPs to the PRS/PGS’es of p<1. At p<1, PGS-Cog and PRS-LOAD showed a correlation coefficient of -0.034 (t=-3.1(8117), p= 0.0022). For comparisons, the effect of the two polygenic scores on level and slope of the cognitive composite score in subsequently non-demented individuals are also presented for six additional p-value thresholds (**Table S4).** Generally, effect sizes of the genetic scores decreased with lower p-value threshold. PRS-LOAD was significantly predictive of slope down to p<0.01, and PGS-Cog remained highly significant also at the lowest threshold tested, p<0.0001.

**Supplementary tables**

**Table S1.** Distribution of individuals included in the study from the eleven age-bins used at inclusion by time (years from inclusion). Drop-out by age-bins can be read diagonally, as there were five year between both time points and age bins. All individuals included were confirmed as non-demented after the study end.

|  | **Age** |  |  |  |  |  |  |  |  |  |  |  |  |  |  |
| --- | --- | --- | --- | --- | --- | --- | --- | --- | --- | --- | --- | --- | --- | --- | --- |
| **Time** | **35** | **40** | **45** | **50** | **55** | **60** | **65** | **70** | **75** | **80** | **85** | **90** | **95** | **100** |  |
| **0** | 57 | 126 | 124 | 118 | 122 | 119 | 103 | 91 | 98 | 86 | 43 | 0 | 0 | 0 | **1087** |
| **5** | 0 | 54 | 120 | 118 | 109 | 118 | 115 | 94 | 83 | 84 | 70 | 20 | 0 | 0 | **985** |
| **10** | 0 | 0 | 52 | 111 | 111 | 102 | 111 | 105 | 81 | 63 | 43 | 43 | 5 | 0 | **827** |
| **15** | 0 | 0 | 0 | 46 | 93 | 92 | 91 | 95 | 73 | 45 | 38 | 23 | 15 | 0 | **611** |
| **20** | 0 | 0 | 0 | 0 | 38 | 71 | 68 | 69 | 64 | 47 | 17 | 14 | 6 | 3 | **397** |
| **25** | 0 | 0 | 0 | 0 | 0 | 26 | 24 | 27 | 24 | 25 | 12 | 3 | 2 | 0 | **143** |
|  | **57** | **180** | **296** | **393** | **473** | **528** | **512** | **481** | **423** | **350** | **223** | **103** | **28** | **3** |  |

**Table S2.** Effect of potential covariates on cognitive composite score (cog-comp). N=1,081 individuals that was confirmed to have stayed non-demented up until one year after the last test occasion. Non-significant covariates were subsequently removed from the final model. Age corresponds to age at inclusion. C=principal component for genetic ancestry.

|  | **Estimate** | **SE** | **t** | **p** |  |
| --- | --- | --- | --- | --- | --- |
| (Intercept) | 2.911e-01 | 7.806e-02 | 3.730 | 0.000201 | *** |
| age_inclusion | -5.695e-01 | 2.374e-02 | -23.990 | 2,00E-16 | *** |
| Time_from_Inclusion | -1.974e-02 | 2.571e-03 | -7.679 | 2.23e-14 | *** |
| (age_inclusion)^2) | -1.505e-01 | 2.441e-02 | -6.164 | 9.79e-10 | *** |
| sex | -8.715e-02 | 4.568e-02 | -1.908 | 0.056642 | . |
| C1 | 9.550e-02 | 2.310e-02 | 4.134 | 3.83e-05 | *** |
| C2 | 4.659e-02 | 2.338e-02 | 1.992 | 0.046559 | * |
| C3 | 7.976e-02 | 2.317e-02 | 3.443 | 0.000597 | *** |
| C4 | 8.533e-03 | 2.309e-02 | 0.369 | 0.711824 |  |
| C5 | -3.390e-02 | 2.299e-02 | -1.475 | 0.140542 |  |
| C6 | -4.720e-02 | 2.300e-02 | -2.052 | 0.040403 | * |
| C7 | -2.870e-02 | 2.316e-02 | -1.239 | 0.215632 |  |
| C8 | -1.035e-02 | 2.350e-02 | -0.440 | 0.659684 |  |
| C9 | 2.739e-02 | 2.309e-02 | 1.186 | 0.235870 |  |
| C10 | -3.507e-03 | 2.290e-02 | -0.153 | 0.878315 |  |
| ApoE ɛ4 | -1.755e-02 | 5.179e-02 | -0.339 | 0.734784 |  |
| ApoE ɛ2 | -7.548e-02 | 6.197e-02 | -1.218 | 0.223424 |  |
| PGS-Cog | 1.696e-01 | 2.309e-02 | 7.347 | 3.84e-13 | *** |
| PRS-LOAD | -1.933e-02 | 2.323e-02 | -0.832 | 0.405561 |  |
| age*Time | -1.623e-02 | 1.214e-03 | -13.377 | 2,00E-16 | *** |
| age^2*TIme | 2.045e-03 | 1.074e-03 | 1.903 | 0.057123 | . |
| Sex*Time | -2.885e-03 | 1.529e-03 | -1.887 | 0.059271 | . |
| C1*Time | 8.268e-04 | 7.474e-04 | 1.106 | 0.268707 |  |
| C2*Time | 6.910e-04 | 7.449e-04 | 0.928 | 0.353629 |  |
| C3*Time | -9.150e-04 | 7.674e-04 | -1.192 | 0.233217 |  |
| C4*Time | -1.005e-04 | 7.787e-04 | -0.129 | 0.897322 |  |
| C5*Time | 2.004e-04 | 7.553e-04 | 0.265 | 0.790799 |  |
| C6*Time | 1.019e-03 | 7.697e-04 | 1.324 | 0.185673 |  |
| C7*Time | 6.860e-04 | 7.791e-04 | 0.881 | 0.378661 |  |
| C8*Time | 1.313e-03 | 7.667e-04 | 1.713 | 0.086893 | . |
| C9*Time | 1.070e-03 | 7.730e-04 | 1.384 | 0.166430 |  |
| C10*Time | -1.291e-04 | 7.454e-04 | -0.173 | 0.862546 |  |
| Apoe ɛ4*Time | -3.408e-03 | 1.692e-03 | -2.015 | 0.044026 | * |
| APoe ɛ2*Time | 1.956e-03 | 2.090e-03 | 0.936 | 0.349550 |  |
| PGS-Cog *TIme | -3.143e-04 | 7.916e-04 | -0.397 | 0.691352 |  |
| PRS-LOAD*Time | -1.757e-03 | 7.693e-04 | -2.284 | 0.022463 | * |

**Table S3**. Descriptive statistics of individual test in subsequently non-demented individuals. SD=standard deviation.

| Test | **T1**  **(N=523)** | **T2**  **(N=1046)** | | | **T3**  **(N=827)** | | | **T4**  **(N=648)** | | | | **T5**  **(N=454)** | | | **T6**  **(N=275)** | | |  |
| --- | --- | --- | --- | --- | --- | --- | --- | --- | --- | --- | --- | --- | --- | --- | --- | --- | --- | --- |
|  | Mean SD | Mean  SD | | | Mean  SD | | | Mean SD | | | Mean  SD | | | | Mean  SD | | |  |
| Episodic recall | 34.77 9.67 | 34.1  11.34 | | | 36.02  10.76 | | | 36,91 10.92 | | | 37.15 10.43 | | | | 38.25  10.43 | | |  |
| Vocabulary | 21.41 5.17 | 21.64  5.55 | | | 22.42  5.13 | | | 22.81 4.82 | | | 23.34  4.59 | | | | 23.45  4 | | |  |
| Block design | 27.0 10.63 | 26.31  10.72 | | | 26.2  10.93 | | | 26.99 10.04 | | | 26.84 10.05 | | | | 26.23  9.84 | | |  |
| Fluency | 21.12 8.09 | 21.21  8.75 | | | 21.38  8.25 | | | 22.73  9.05 | | | 23.31  8.4 | | | | 24.1  9.15 | | |  |
|  |  |  |  |  | |  |  | |  |  | | |  |  | |  |  | |

**Table S4.** Effect of genetic predictors on intercept and slope of individual cognitive tests, including all six test occasions.

|  | **Estimate** | **SE** | **t** | **p** |  |
| --- | --- | --- | --- | --- | --- |
| **Fluency** |  |  |  |  |  |
| ApoE ɛ4 | -7.259e-02 | 4.977e-01 | -0.146 | 0.8841 |  |
| **PGS-Cog** | 1.291e+00 | 2.227e-01 | 5.797 | 8.37e-09 | *** |
| **PRS-LOAD** | -2.185e-01 | 2.234e-01 | -0.978 | 0.3282 |  |
| Time*ApoE ɛ4 | -1.325e-02 | 2.448e-02 | -0.541 | 0.5884 |  |
| **Time*PGS-Cog** | -1.266e-02 | 1.142e-02 | -1.108 | 0.2678 |  |
| **Time*PRS-LOAD** | -2.341e-02 | 1.115e-02 | -2.100 | 0.0358 | * |
|  |  |  |  |  |  |
| **Episodic memory** |  |  |  |  |  |
| ApoE ɛ4 | -1.932e-01 | 5.461e-01 | -0.354 | 0.72353 |  |
| **PGS-Cog** | 1.071e+00 | 2.449e-01 | 4.373 | 1.32e-05 | *** |
| **PGS-LOAD** | -1.516e-01 | 2.450e-01 | -0.619 | 0.53615 |  |
| Time*ApoE ɛ4 | -4.251e-02 | 2.708e-02 | -1.570 | 0.11655 |  |
| **Time*PGS-Cog** | -1.261e-02 | 1.268e-02 | -0.995 | 0.31995 |  |
| **Time*PRS-LOAD** | -1.750e-02 | 1.233e-02 | -1.419 | 0.15588 |  |
|  |  |  |  |  |  |
| **Vocabulary** |  |  |  |  |  |
| ApoE ɛ4 | -5.958e-03 | 3.249e-01 | -0.018 | 0.9854 |  |
| **PGS-Cog** | 9.236e-01 | 1.450e-01 | 6.369 | 2.72e-10 | *** |
| **PRS-LOAD** | -1.623e-01 | 1.458e-01 | -1.113 | 0.2658 |  |
| Time*ApoE ɛ4 | -1.145e-02 | 1.112e-02 | -1.030 | 0.3032 |  |
| **Time*PGS-Cog** | 2.075e-03 | 5.188e-03 | 0.400 | 0.6892 |  |
| **Time*PRS-LOAD** | 2.687e-03 | 5.097e-03 | 0.527 | 0.5982 |  |
|  |  |  |  |  |  |
| **Blockdesign** |  |  |  |  |  |
| ApoE ɛ4 | -8.502e-02 | 5.607e-01 | -0.152 | 0.879512 |  |
| **PGS-Cog** | 1.361e+00 | 2.508e-01 | 5.428 | 6.80e-08 | *** |
| **PRS-LOAD** | -1.371e-02 | 2.513e-01 | -0.055 | 0.956497 |  |
| Time*ApoE ɛ4 | -3.364e-02 | 2.461e-02 | -1.367 | 0.171728 |  |
| **Time*PGS-Cog** | 1.414e-02 | 1.154e-02 | 1.226 | 0.220308 |  |
| **Time*PRS-LOAD** | -2.343e-02 | 1.121e-02 | -2.090 | 0.036695 | * |

**Table S5.** Effect of intercept and slope on the cognitive performance composite score by polygenic scores for cognitive ability and Alzheimer’s disease calculated with different p-value thresholds.

| **p<0.5** | **Estimate** | **SE** | **t** | **p** |  |
| --- | --- | --- | --- | --- | --- |
| PGS-Cog | 1.669e-01 | 2.302e-02 | 7.251 | **7.53e-13** | *** |
| PRS-LOAD | -1.814e-02 | 2.314e-02 | -0.784 | 0.433289 |  |
| PGS-Cog*Time | -4.402e-04 | 7.862e-04 | -0.560 | 0.575603 |  |
| PRS-LOAD*Time | -1.868e-03 | 7.667e-04 | -2.437 | **0.014867** | * |
|  |  |  |  |  |  |
| **p<0.1** |  |  |  |  |  |
| PGS-Cog | 1.672e-01 | 2.303e-02 | 7.263 | **6.95e-13** | *** |
| PRS-LOAD | -2.949e-02 | 2.320e-02 | -1.271 | 0.204017 |  |
| PGS-Cog*Time | 2.232e-04 | 7.783e-04 | 0.287 | 0.774262 |  |
| PRS-LOAD*Time | -1.546e-03 | 7.554e-04 | -2.047 | **0.040715** | * |
|  |  |  |  |  |  |
| **p<0.05** |  |  |  |  |  |
| PGS-Cog | 1.560e-01 | 2.284e-02 | 6.827 | **1.40e-11** | *** |
| PRS-LOAD | -2.645e-02 | 2.362e-02 | -1.120 | 0.26297 |  |
| PGS-Cog*Time | -5.998e-04 | 7.540e-04 | -0.795 | 0.42643 |  |
| PRS-LOAD*Time | -1.481e-03 | 7.487e-04 | -1.978 | **0.04803** | * |
|  |  |  |  |  |  |
| **p<0.01** |  |  |  |  |  |
| PGS-Cog | 1.588e-01 | 2.285e-02 | 6.951 | **6.07e-12** | *** |
| PRS-LOAD | -5.789e-02 | 2.332e-02 | -2.482 | **0.013205** | * |
| PGS-Cog*Time | -3.769e-04 | 7.544e-04 | -0.500 | 0.617382 |  |
| PRS-LOAD*Time | -1.508e-03 | 7.507e-04 | -2.008 | **0.044721** | * |
|  |  |  |  |  |  |
| **p<0.001** |  |  |  |  |  |
| PGS-Cog | 1.182e-01 | 2.295e-02 | 5.151 | **3.05e-07** | *** |
| PRS-LOAD | -4.690e-02 | 2.435e-02 | -1.926 | 0.054337 | . |
| PGS-Cog*Time | -9.482e-04 | 7.358e-04 | -1.289 | 0.197587 |  |
| PRS-LOAD*Time | -3.210e-04 | 7.795e-04 | -0.412 | 0.680477 |  |
|  |  |  |  |  |  |
| **p<0.0001** |  |  |  |  |  |
| PGS-Cog | 1.108e-01 | 2.303e-02 | 4.814 | **1.68e-06** | *** |
| PRS-LOAD | -3.691e-02 | 2.677e-02 | -1.379 | 0.16827 |  |
| PGS-Cog*Time | -3.053e-04 | 7.338e-04 | -0.416 | 0.67739 |  |
| PRS-LOAD*Time | -7.961e-05 | 8.744e-04 | -0.091 | 0.92747 |  |

**Supplementary figures**

**Figure S1, A-D**. Spaghetti plots of individual trajectories in test performance over six test occasions for individual cognitive test.


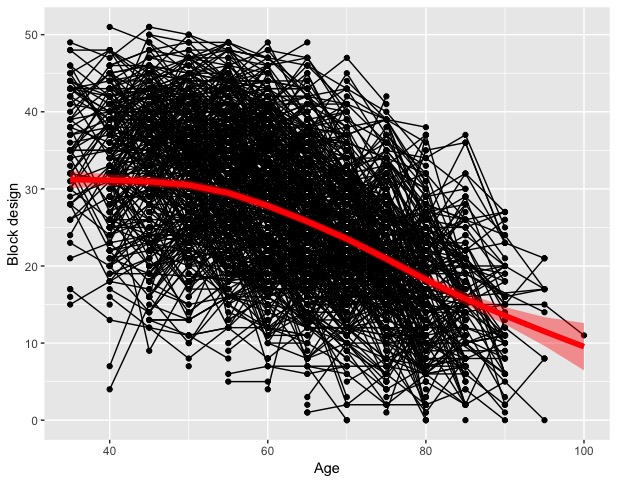

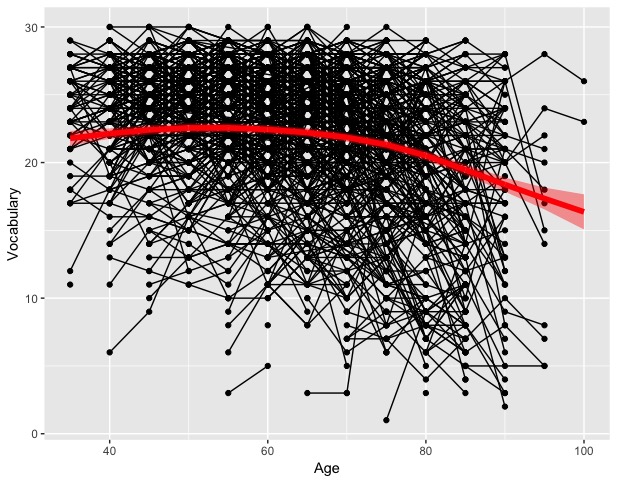

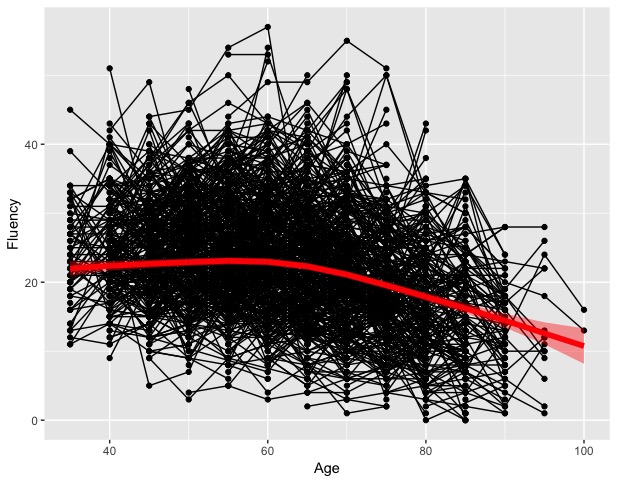

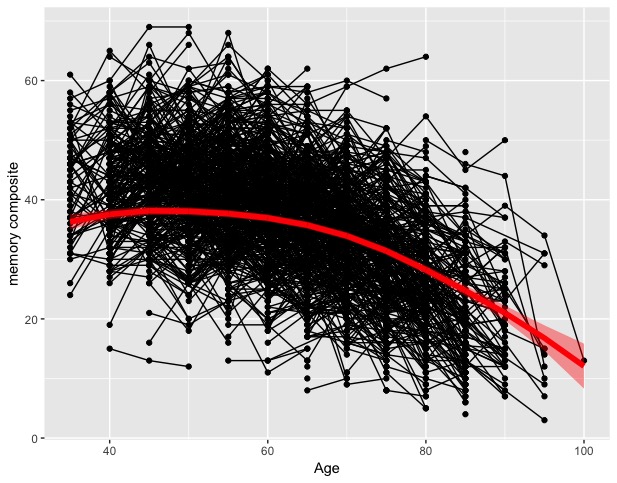

Supplement: Supplementary file 1 — Supplementary materials [file 41398_2020_934_MOESM1_ESM.docx]
